# Hydrocortisone for prevention or treatment of bronchopulmonary dysplasia: long-term neurodevelopmental safety and efficacy—a meta-analysis of randomized clinical trials

**DOI:** 10.3389/fped.2026.1851870

**Published:** 2026-06-24

**Authors:** Kawthar Ahmed Shehab, Rahaf Muslih N. Almatrafi, Ethar Ali, Yara Albargi, Sama Saabi, Halah Alhassan Aljohani, Rayan Nabil Almuhanna, Raghad Alnaami, Khalid Abdulrahman Almehery, Reema Safar Alamri, Sarah Ahmed A. Baqer, Shouq Naif Aloufi, Ahmed Elaraby, Bedah Mohammed Alnawfal

**Affiliations:** 1 College of Medicine, Alfaisal University, Riyadh, Saudi Arabia; 2 College of Medicine, Northern Border University, Arar, Saudi Arabia; 3 College of Medicine, Ahfad University for Women, Omdurman, Sudan; 4 College of Medicine, Jazan University, Jazan, Saudi Arabia; 5 College of Medicine, Istanbul University, Istanbul, Türkiye; 6 College of Medicine, Imam Abdulrahman Bin Faisal University, Dammam, Eastern Province, Saudi Arabia; 7 College of Medicine, King Abdulaziz University, Jeddah, Saudi Arabia; 8 Abha Maternity and Children Hospital, Abha, Saudi Arabia; 9 College of Medicine, Jordan University of Science and Technology, Irbid, Jordan; 10 College of Medicine, Taibah University, Madinah, Saudi Arabia; 11 Faculty of Medicine, Al-Azhar University, Cairo, Egypt; 12 Department of Pediatrics Hematology and Oncology, King Saud Medical City, Riyadh, Saudi Arabia

**Keywords:** bronchopulmonary dysplasia, hydrocortisone, meta-analysis, preterm infants, safety

## Abstract

**Background:**

Postnatal systemic steroids can reduce inflammation that contributes to bronchopulmonary dysplasia (BPD) in extremely preterm infants; however, prior experience with dexamethasone raised concerns regarding long-term neurodevelopmental harm. As a result, hydrocortisone has been proposed as a potentially safer alternative.

**Methods:**

We conducted a meta-analysis of randomized controlled trials comparing hydrocortisone with placebo/routine care in preterm infants, stratified by early prophylaxis (<7 days) or late treatment (≥7 days). We searched major databases through February 2026. The primary outcome was the composite of death or neurodevelopmental impairment (NDI) at 2 years of corrected age.

**Results:**

We included 17 studies representing seven distinct trials and their follow-up publications, comprising a total of 2,213 infants (1,096 receiving hydrocortisone and 1,117 receiving placebo). At 2 years, death/NDI occurred in 53.5% (531/993) of infants in the hydrocortisone group and 56.8% (581/1,023) of infants in the placebo group, with no significant effect observed with either early administration [risk ratio (RR) 0.89, 95% CI 0.70–1.13; *I*²=0%] or late administration (RR 0.97, 95% CI 0.80–1.17; *I*²=0%). Hydrocortisone was not associated with significant differences in death at 2 years (17.1% vs. 20.8%) or NDI at 2 years (42.5% vs. 43.9%) nor in cerebral palsy, hearing impairment, or visual impairment. Exploratory analyses showed no significant reduction in death or BPD at 36 week of postmenstrual age overall, and available school-age outcomes did not demonstrate differences in neurocognitive measures.

**Conclusion:**

Across randomized trials with long-term follow-up, postnatal hydrocortisone was not associated with improved survival free of NDI at 2 years nor was it associated with an increased risk of major neurodevelopmental or sensory impairments. These findings do not demonstrate an increased neurodevelopmental risk with hydrocortisone as used in current regimens, but its use should still be individualized based on respiratory phenotype, timing, and short-term monitoring for steroid-related adverse effects.

**Systematic Review Registration:**

PROSPERO CRD420261280132

## 1 Introduction

Bronchopulmonary dysplasia (BPD) is the most serious complication affecting extremely preterm infants, with incidence rates rising despite advances in surfactant therapy and non-invasive ventilation. The incidence rate of BPD ranges between 26% and 40% among infants born at extremely low gestational ages (1, 2). Preterm birth below 28 week' of gestation is frequently associated with systemic inflammation, which is a major risk factor for BPD, brain damage, and subsequent neurodevelopmental disorders (2, 3). Thus, therapies modulating this inflammation are critical for improving both respiratory and neurological outcomes.

Systemic corticosteroids have been suggested as the most potent anti-inflammatory agents. Until the early 2000s, dexamethasone was widely used, as clinical trials showed its benefits in reducing the risk of death or BPD (4, 5). However, this procedure was questioned, and its use was decreased after landmark trials outweighed the dexamethasone benefit on survival without BPD by an increased risk of neurodevelopmental impairment (NDI), specifically cerebral palsy (CP) and other neurodevelopmental disorders, forcing the clinicians to trade respiratory survival for neurodevelopmental safety (6).

Hydrocortisone was proposed by Watterberg and colleagues as a safer option to maintain the respiratory benefits of glucocorticoids while avoiding their adverse effects on the developing brain (7). However, the first clinical trials of hydrocortisone treatment to prevent BPD were inconclusive (8–10), which warranted larger trials such as the PREMILOC and STOP-BPD (11, 12). The PREMILOC trial demonstrated a significant reduction in the survival with BPD among extremely preterm infants. In contrast, the STOP-BPD trial found no significant difference between hydrocortisone and placebo in survival without BPD at 36 weeks of postmenstrual age.

In this systematic review and meta-analysis of randomized controlled trials, we aimed to synthesize the evidence on the use of hydrocortisone in preventing BPD among infants born before 28 weeks of gestation and provide the clinical setting with the most updated, collective, and conclusive assessment of both the benefits and adverse effects of this intervention, especially neurodevelopmental outcomes including the longest follow-up trials.

## 2 Methods

We conducted this systematic review and meta-analysis in accordance with the Preferred Reporting Items for Systematic Reviews and Meta-Analyses (PRISMA) statement guidelines (13) and the methodologies of the Cochrane Handbook for Systematic Reviews of Interventions (14). The study was registered on PROSPERO (CRD420261280132).

### 2.1 Literature search

We searched PubMed, Scopus, Web of Science (WoS), Embase, and Cochrane CENTRAL from inspection until February 2026 using the following search term: (hydrocortisone OR cortisol) AND (preterm OR premature OR newborn OR neonate OR infant OR low birth weight OR VLBW OR ELBW) AND (bronchopulmonary dysplasia OR BPD OR chronic lung disease). Detailed search strategies for each database are reported in Supplementary Table 1. All citations were imported into Zotero, duplicates were removed, and a manual backward citation analysis was conducted for all references to include all possible relevant reports.

### 2.2 Eligibility criteria

**Figure 1:**
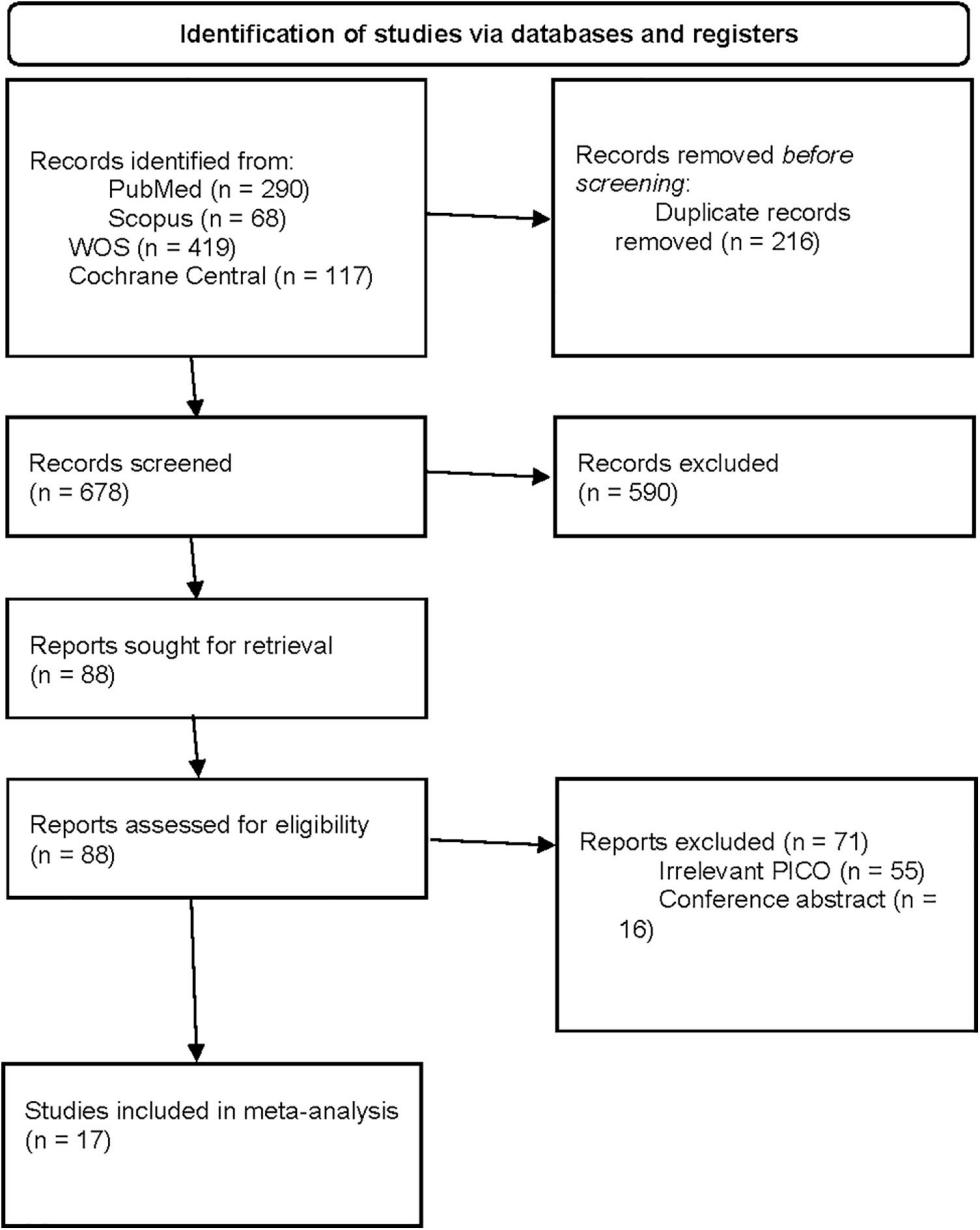
PRISMA flow diagram.

We screened the results in a two-step approach. Two reviewers (KS, RahA) independently screened all records in a two-stage process. In Stage 1, titles and abstracts of all retrieved records were screened against the eligibility criteria following the removal of duplicate records. In Stage 2, the full texts of records passing Stage 1 were assessed against the same criteria, and reasons for exclusion at the full-text stage were recorded and presented in the PRISMA flow diagram (Figure 1). Reviewers were blinded to each other's decisions; disagreements were initially resolved through discussion, and unresolved disagreements were arbitrated by a third senior reviewer (AE). Data extraction was performed independently by two reviewers using an extraction sheet and was subsequently cross-checked for accuracy. We included only randomized controlled trials that compared hydrocortisone with conventional routine care in preterm neonates for the prevention of BPD, focusing on the long-term neurodevelopmental outcomes. The studies had to report the outcomes of interest, with the primary outcome being a composite of NDI and death. We also included NDI, death, CP, hearing and visual impairment, and other secondary outcomes. Studies were excluded at the title/abstract stage if they (i) were not conducted in humans, (ii) did not include preterm or low-birth-weight infants, (iii) did not evaluate systemic hydrocortisone (evaluated inhaled, topical, or non-hydrocortisone corticosteroids only), (iv) were not randomized trials (observational, quasi-experimental, single-arm, or modeling studies), or (v) were duplicate publications without new outcome data. At the full-text stage, additional reasons for exclusion were: (a) head-to-head comparison against another corticosteroid (hydrocortisone vs. dexamethasone) with no placebo or routine-care arm; (b) failure to report any of the prespecified outcomes; (c) conference abstracts, letters, editorials, narrative reviews, or case reports; (d) published in a language other than English; and (e) full text not retrievable despite author contact.

### 2.3 Outcome definitions

NDI was extracted as defined by each trial. Across the included trials, NDI definitions used the Bayley Scales of Infant Development, second edition (BSID-II) or third edition (BSID-III), with cognitive/motor cutoffs ranging from <70 to <85, in combination with cerebral palsy [with or without gross motor function classification system (GMFCS) grading], bilateral hearing impairment requiring amplification, and bilateral severe visual impairment; some trials also included behavioral outcomes. The full definition used by each trial is summarized in Table 1. When a trial reported multiple severity thresholds, we extracted the moderate-to-severe NDI definition (typically a Bayley score <70, CP with GMFCS level ≥2, and bilateral sensory impairment).

**Table 1:** Definition of neurodevelopmental impairment (NDI) in each included trial.

Trial and follow-up	Timing (early <7 days/late ≥7 days)	Age at assessment	Cognitive tool and cutoff	Motor/CP definition	Overall NDI severity grading
Baud 2017/2019 (PREMILOC 2 years)	Early	22–26 months of CA	Revised Brunet–Lézine (RBL) scale; global developmental quotient; cutoffs: DQ <70 = moderate–severe; 70–84 = mild; ≥85 = no NDI	Cerebral palsy, per the Executive Committee for the Definition of Cerebral Palsy; GMFCS not used to grade	Three-tier: no NDI/mild/moderate-to-severe NDI
Halbmeijer 2021 (STOP-BPD 2 years)	Late	2 years of CA	Bayley-III-NL (Dutch) Cognitive composite <85 OR motor composite <85	Cerebral palsy with GMFCS > level II	Dichotomous (NDI present vs. absent)
Watterberg 2022 (NRN/NEJM 2 years)	Late	22–26 months of CA	Bayley-III Cognitive composite <85 OR motor composite <85	GMFCS level ≥ II	Dichotomous, labeled “moderate or severe NDI”
Watterberg 2007 (early HC pilot 2 years)	Early	18–22 months of CA	Bayley-II (BSID-II) MDI <70 OR PDI <70 (≥2 SD below mean)	Cerebral palsy diagnosed clinically (Bax/Palisano framework); GMFCS not used to grade severity	Dichotomous
Peltoniemi 2008 (Peltoniemi 2 years)	Early	2 years of CA	Bayley-II (BSID-II) MDI <70 (or DQ <70 when Bayley not feasible)	Cerebral palsy diagnosed by a pediatric neurologist or pediatrician; GMFCS not used to grade severity	Dichotomous (severe NDI)
Parikh 2015 (stress-dose HC 2 years)	Late	18–22 months of CA	Bayley-III Cognitive score <80 OR language score <80 (note: <80 cutoff, not <70 or <85)	Cerebral palsy = ≥ 2 of: delayed motor milestones, abnormal neuromotor exam, or aberrant primitive reflexes; GMFCS not used to grade severity	Dichotomous
Trousson 2023 (PREMILOC 5 years)	Early	5 years	WPPSI (Dutch); NEPSY Full-scale IQ < 70 used as a composite component	Cerebral palsy graded with GMFCS (levels I, II–III, >III reported)	Composite: CP + ASD + FSIQ < 70
Peltoniemi 2015 (early HC school age)	Early	5–7 years	WPPSI-R (verbal IQ, performance IQ, full-scale IQ); RDLS-III for language	Cerebral palsy graded per Bax (“grades of CP”); GMFCS not used	Graded as minor neurological dysfunction → severe neurological condition (e.g., CP); no fixed multitier composite
de Baat 2026 (STOP-BPD 5.5 years)	Late	5.5 years of CA	WPPSI (Dutch); M-ABC-2-NL FSIQ thresholds: >85, 70–85, 55–70, <55	Cerebral palsy + GMFCS levels 1, 2–3, 4–5 used for severity	Four-tier: no NDI/mild/moderate/severe NDI
DeMauro 2025 (NRN school age)	Late	5 years 0 months–7 years 11 months	DAS-II; GCA score Cognitive delay = GCA < 70 (≥2 SD below mean of 100)	Motor delay = moderate–severe CP with GMFCS level II–V, or M-ABC; total impairment score ≤ 5th percentile	Composite “functional impairment” = any of: cognitive delay, motor delay, academic delay, exercise-capacity delay

ASD, autism spectrum disorder; Bayley-II, Bayley scales of infant development, second edition; Bayley-III, Bayley scales of infant and toddler development, third edition; BSID, Bayley scales of infant development; CA, corrected age; CP, cerebral palsy; DAS-II, differential ability scales, second edition; DQ, developmental quotient; ENT, ear–nose–throat; FSIQ, full-scale intelligence quotient; GCA, global conceptual ability; GMFCS, gross motor function classification system; HC, hydrocortisone; MDI, mental development index; M-ABC-2-NL, movement assessment battery for children, second edition, Dutch version; NDI, neurodevelopmental impairment; NEPSY, a developmental NEuroPSYchological assessment; PDI, psychomotor development index; RBL, revised Brunet–Lézine; RDLS-III, Reynell developmental language scale III; SD, standard deviation; WPPSI/WPPSI-R, Wechsler preschool and primary scale of intelligence/revised.

### 2.4 Quality assessment

We assessed the risk of bias of the included studies using the Cochrane Risk of Bias 2 (ROB-2) tool (15). The tool evaluates five domains: (1) bias arising from the randomization process, (2) bias due to deviations from intended interventions, (3) bias due to missing outcomes data, (4) bias in the measurement of outcomes, and (5) bias in the selection of reported results. The decision for each domain was rated as either high risk, some concerns, or low risk, and an overall risk of bias was assigned based on each domain judgment. Any disagreements between the authors were resolved via discussion.

### 2.5 Data extraction

We used a standardized Excel sheet to extract data from the included studies. The extracted data comprised four main categories: (a) baseline characteristics of the included patients, (b) summary characteristics of the included studies, (c) the Newcastle-Ottawa Scale (NOS) domains, and (d) outcome measures.

To prevent double-counting participants, we mapped every included publication to its parent trial and treated each randomized cohort as a single unit. For trials with more than one publication, we used a single prespecified source per outcome (the publication providing the most complete and longest follow-up data for that outcome) and did not pool numerical data across multiple reports of the same outcome from the same trial. When several publications reported the same outcome and time point, we used the most complete report with the longest follow-up. To ensure that no infants were counted twice, two reviewers manually verified extracted data. The complete trial-to-publication mapping is provided in Table 2.

**Table 2:** Summary of the characteristics of the included studies.

Trial name	Associated reports	Country	Duration	Total sample size	Patient characteristics	Inclusion criteria	Exclusion criteria	Intervention	Control	Outcomes assessed	Key findings	Follow-up
STOP-BPD	Onland 2019, Halbmeijer 2021, Halbmeijer 2024, Baat 2025	Netherlands, Belgium	Nov 2011–Dec 2016	*N* = 372	Very preterm infants, ventilator-dependent	GA <30 weeks and/or BW <1,250 g, ventilator-dependent	Chromosomal defects, major congenital malformations, prior (first week) postnatal steroids for lung function	Hydrocortisone: 22-day tapering course (cumulative 72.5 mg/kg)	Placebo	Death or BPD at 36 weeks of PMA, short-term morbidity	No significant difference in death or BPD (70.7% HC vs. 73.7% placebo)	2–5.5 years
NICHD NRN	Watterberg 2022, DeMauro 2025	USA (19 centers)	Aug 2011–Feb 2018	*N* = 800	Mean GA: 24.9 weeks; mean BW: 715 g; 100% intubated	GA <30 weeks; intubated for ≥7 days; intubated at randomization (14–28 days)	Major anomalies; prior systemic steroids; limiting life support	HC: 4 mg/kg/day tapered over 10 days (total ∼17 mg/kg)	Placebo	Survival w/o BPD, safety, survival w/o NDI	No diff in survival w/o BPD (16.6% HC vs. 13.2% Placebo).	22–26 months of CA, 5–7 years (school)
PREMILOC	Baud 2016, Baud 2017, Baud 2019, Trousson 2022	France (21 NICUs)	May 2008–Jan 2014	*N* = 523	Mean GA: 26 weeks; mean BW: ∼885 g; all inborn	Born <28 weeks of GA; <24 h old; inborn	ROM <22 weeks; severe IUGR; severe asphyxia; major malformations	HC: 0.5 mg/kg q12 h×7 days, then q24 h×3 days (total 10 days), started <24 h	Placebo	Survival w/o BPD at 36 weeks, mortality, neurodevelopment	Survival w/o BPD significantly higher in HC (60% vs. 51%, p = 0.04)	22 months of CA, 5 years
Watterberg	Watterberg 2004, Watterberg 2007	USA (9 centers)	Nov 2001–Apr 2003	*N* = 360	Mean GA: 25.3 weeks; mean BW: ∼730 g	BW 500–999 g; ventilated at 12–48 h	Major anomaly; congenital sepsis; postnatal steroids; triplets+	HC: 1 mg/kg/day×12 days, then 0.5 mg/kg/day×3 days (total 15 days)	Placebo	Survival w/o BPD at 36 weeks, mortality, growth	No difference in BPD outcome	18–22 months of CA
Parikh	Parikh 2015	USA (single center)	Nov 2005–Sep 2008	*N* = 64	Mean GA: 25 weeks; mean BW: ∼670 g; high-risk BPD	BW <1,000 g; age 10–21 days; ventilator-dependent; respiratory index >2	Major congenital anomalies	HC: 3 mg/kg/day tapering over 7 days	Placebo	Composite death or NDI at 18–22 months	No significant difference in death or NDI (68% HC vs. 76% placebo)	18–22 months of CA
Peltoniemi	Peltoniemi 2008, Peltoniemi 2015	Finland	2002	*N* = 51	Mean GA: 27 weeks; mean BW: ∼925 g	BW 501–1,250 g; GA 23–30w; ventilated <24 h	Lethal malformations; likely early death	HC: 2 mg/kg/day tapering over 10 days	Placebo	BPD, CP, NDI	Trial stopped early due to GI perforations (40% in HC vs. 4% placebo)	2 years of CA, 5–7 vears
Bonsante	Bonsante 2007	Italy	April 2003 - Sep 2005	*N* = 50	Mean GA ∼26 weeks; BW ∼900 g	Birth weight 500–1,250 g; mechanical ventilation; informed consent	Major congenital malformations; hydrops fetalis; maternal steroids >2 courses	Hydrocortisone: 0.5 mg/kg q12 h × 9days, then 0.5 mg/kg q24 h × 3 days *(cumulative: ∼10.5 mg/kg)*	Placebo	Survival w/o CLD at 36 weeks	No significant difference in survival without CLD (64% HC vs. 44% placebo)	36 weeks of PMA

GA, gestational age; BW, birth weight; BPD, bronchopulmonary dysplasia; HC, hydrocortisone; NDI, neurodevelopmental impairment; w/o, without; CP, cerebral palsy; CLD, chronic lung disease.

Dichotomous data were extracted as the number of events and the total number of included patients in each study. Pooled risk ratios (RRs) for dichotomous data and mean/standardized mean differences for continuous data, each with 95% CIs, were calculated using a random-effects model with the REML estimator and Hartung–Knapp-adjusted confidence intervals; the DerSimonian–Laird estimator was used as a sensitivity analysis. We also qualitatively assessed clinical and methodological heterogeneity across the dimensions of timing of intervention (early prophylaxis vs. late treatment), cumulative hydrocortisone dose, target population (all preterm vs. ventilator-dependent infants), and outcome definitions. In addition, we reported a DOI plot and the LFK index to test for possible publication bias.

### 2.6 Sensitivity analyses

We prespecified two sensitivity analyses: (i) leave-one-out analyses, in which each trial was sequentially omitted and the analysis was repeated for the primary outcome (death or NDI at 2 years of corrected age) and key secondary outcomes (NDI alone, death alone, BPD at 36 weeks of postmenstrual age, and the composite of death or BPD at 36 weeks); and (ii) analyses were restricted to trials enrolling ≥100 infants to assess the effect of smaller pilot trials. All data analyses were performed using RStudio and Stata/MP version 19 (StataCorp LLC, Texas, USA).

## 3 Results

### 3.1 Literature search

Our comprehensive search yielded 894 citations; 806 records were excluded after duplicate removal and title/abstract screening, leaving 88 publications for full-text screening. Of these, 17 reports were finally included (1, 8–12, 16–27). These publications represented seven distinct trials (8–12, 22, 27) and their follow-up papers. The selection process is summarized in the PRISMA flow diagram (Figure 1).

### 3.2 Study characteristics and risk-of-bias assessment

The finally included randomized controlled trials (RCTs) were conducted across multiple centers worldwide. The studies included a total of 2,213 patients; of these, 1,096 (49.5%) patients were allocated to receive hydrocortisone and 1,117 (50.4%) were assigned to the placebo group. Four studies used early hydrocortisone administration for prophylaxis (<7 days after birth), comprising 984 (44.5%) patients (8–11), and the other three used late administration for treatment (>7 days after birth), comprising 1,229 (55.5%) patients (12, 22, 27). Among the total population, 1,160 (52.4%) were boys and 1,053 (47.6%) were girls, with a mean gestational age of 25.5 weeks. Six trials reported outcomes at 2 years of corrected age (CA) (16, 20, 22, 23, 26, 27), and four trials reported outcomes up to school age (18, 19, 24, 25). Detailed summary and baseline characteristics of the included studies and patients are presented in Tables 2–4. All included RCTs were assessed for risk of bias using the ROB2 tool, as shown in Supplementary Figure 1. Most of the studies were of good quality (10–12, 27). Three studies were rated as having some concerns (8, 9, 22).

**Table 3:** Baseline characteristics of patients included within the studies.

Trial name	Sample size		Maternal age (mean, SD, years)				Gestational age (mean, SD, weeks)				Male (*N*, %)				Birth weight (mean, SD, g)				Multiple births/multiple pregnancies (*N*, %)				Antenatal steroid use (*N*, %)				Chorioamnionitis (*N*, %)				Cesarean delivery (*N*, %)			
			HC		Placebo		HC		Placebo		HC		Placebo		HC		Placebo		HC		Placebo		HC		Placebo		HC		Placebo		HC		Placebo	
	HC	Placebo	Mean/median	SD/IQR	Mean/median	SD/IQR	Mean/median	SD/IQR	Mean/median	SD/IQR	N	%	N	%	Mean/median	SD/IQR	Mean/median	SD/IQR	N	%	N	%	N	%	N	%	N	%	N	%	N	%	N	%
STOP-BPD	182	190	30	27–34	30	27–34	25.4	24.9–26.4	25.6	24.7–26.4	96	52.80%	109	57.40%	775	644–865	710	629–810	70	39%	54	28%	159	87%	172	91%	51	28.00%	53	27.90%	98	54%	111	58%
NICHD NRN	398	402	28.5	6.2	28.2	6.4	24.9	1.5	24.9	1.5	186	46.7	235	58.5	710.3	1.63	720	172	95	24	102	25	343	87	357	89	NR	NR	NR	NR	NR	NR	NR	NR
PREMILOC	255	266	29·9	26·5–34·5	29·9	26·5–34·7	26.4	25.6–27	26.5	25.7–27.1	131	51%	149	56%	860	750–970	840	735–970	82	32%	90	34%	238	93%	246	92%	114	50	127	53	79	31%	70	26
Watterberg	180	180	NR	NR	NR	NR	25.2	1.5	25.3	1.7	96	53%	90	50%	731	126	734	126	42	23%	38	21%	138	77%	146	81%	73	53%	76	52%	103	57%	117	65%
Parikh	28	29	NR	NR	NR	NR	25	24–26	25	24–26	13	45%	17	59%	683	107	658	129	6	21%	8	28%	22	79%	18	62%	NR	NR	NR	NR	NR	NR	NR	NR
Peltoniemi	23	22	NR	NR	NR	NR	26.8	1.5	27.1	1.5	14	61%	12	55%	903	206	949	207	NR	NR	NR	NR	21	91%	21	96%	NR	NR	NR	NR	NR	NR	NR	NR
Bonsante 2007	25	25	NR	NR	NR	NR	26.2	25.2–27.4	26.5	25.0–28.1	13	52%	16	64%	0.84	0.7 –1	0.9	0.7–1.01	NR	NR	NR	NR	13	52%	17	68%	9	36%	13	52%	NR	NR	NR	NR

HC, hydrocortisone; SD, standard deviation; IQR, interquartile range; N, number; NR, not reported.

**Table 4:** Treatment methodology in each trial.

Study ID	Purpose	Timing (postnatal age)	Intervention regimen	BPD definition (primary outcome)	NDI definition (long-term outcome)
PREMILOC (Baud 2016)	Prophylaxis (early)	< 24 h	Low dose: 0.5 mg/kg q12h × 7 days, then q24h × 3 days (total: 8.5 mg/kg over 10 days)	Physiological: oxygen dependency at 36 weeks of PMA; confirmed by the room air challenge if FiO_2_ < 0.30	Composite: CP (GMFCS ≥ 2), Bayley-III score < 85 (−1 SD), blindness, or deafness
Bonsante (Bonsante 2007)	Prophylaxis (early)	< 48 h	Intermediate: 0.5 mg/kg q12h × 9 days, then q24h × 3 days (total: 10.5 mg/kg over 12 days)	Clinical: oxygen dependency at 36 weeks of PMA (no explicit room air challenge reported)	Not reported (no long-term follow-up)
Watterberg 2004	Prophylaxis (early)	12–48 h	Intermediate: 1 mg/kg/day (split q12h) × 12 days, then 0.5 mg/kg/day × 3 days (total: 13.5 mg/kg over 15 days)	Physiological: clinical O_2_ use + physiological confirmation (O_2_ needed for saturation ≥90%)	Composite: CP, Bayley-II MDI or PDI < 70 (−2 SD), blindness, or deafness
Peltoniemi (Peltoniemi 2005)	Prophylaxis (early)	< 36 h	High Initial: 2 mg/kg/day × 2d, 1.5 mg/kg/day × 2 days, 0.75 mg/kg/day × 6days (total: 11.5 mg/kg over 10 days)	Clinical: requirement for supplemental oxygen at 36 weeks of PMA	Composite: CP, IQ < 70 (−2 SD), blindness, deafness, or severe behavioral problems
STOP-BPD (Onland 2019)	Treatment (late)	Days 7–14	High cumulative: 5 mg/kg/day × 7days, tapered over next 15 days (total: 72.5 mg/kg over 22 days)	Physiological (Walsh): O_2_ reduction test if FiO_2_ < 0.30; failure = saturation < 0%	Composite: CP (GMFCS ≥ 2), Bayley-III score < 85 (−1 SD), blindness, or hearing loss
NRN Trial (Watterberg 2022)	Treatment (late)	Days 14–28	Medium cumulative: 4 mg/kg/day × 4 days, 2 mg/kg/day × 3 days, 1 mg/kg/day × 3 days (total: 25 mg/kg over 10 days)	Jensen graded: moderate (O_2_ < 30%) or severe (O_2_ ≥ 30% or PPV); room air challenge used	Composite: CP (GMFCS ≥ 2), Cognitive Score < 85 (−1 SD), Blindness, or Hearing Loss requiring aids
Parikh 2015	Treatment (late)	Days 10–21	Stress dose (Titrated): 15 mg/m²/day (∼1 mg/kg) adjusted to cortisol > 20 µg/dL (total: variable/low over 7 days)	Physiological (Walsh): referenced Walsh criteria for secondary BPD outcome	Composite: CP, Bayley-III score < 70 (−2 SD), blindness, or deafness

### 3.3 Primary outcome

**Figure 2:**
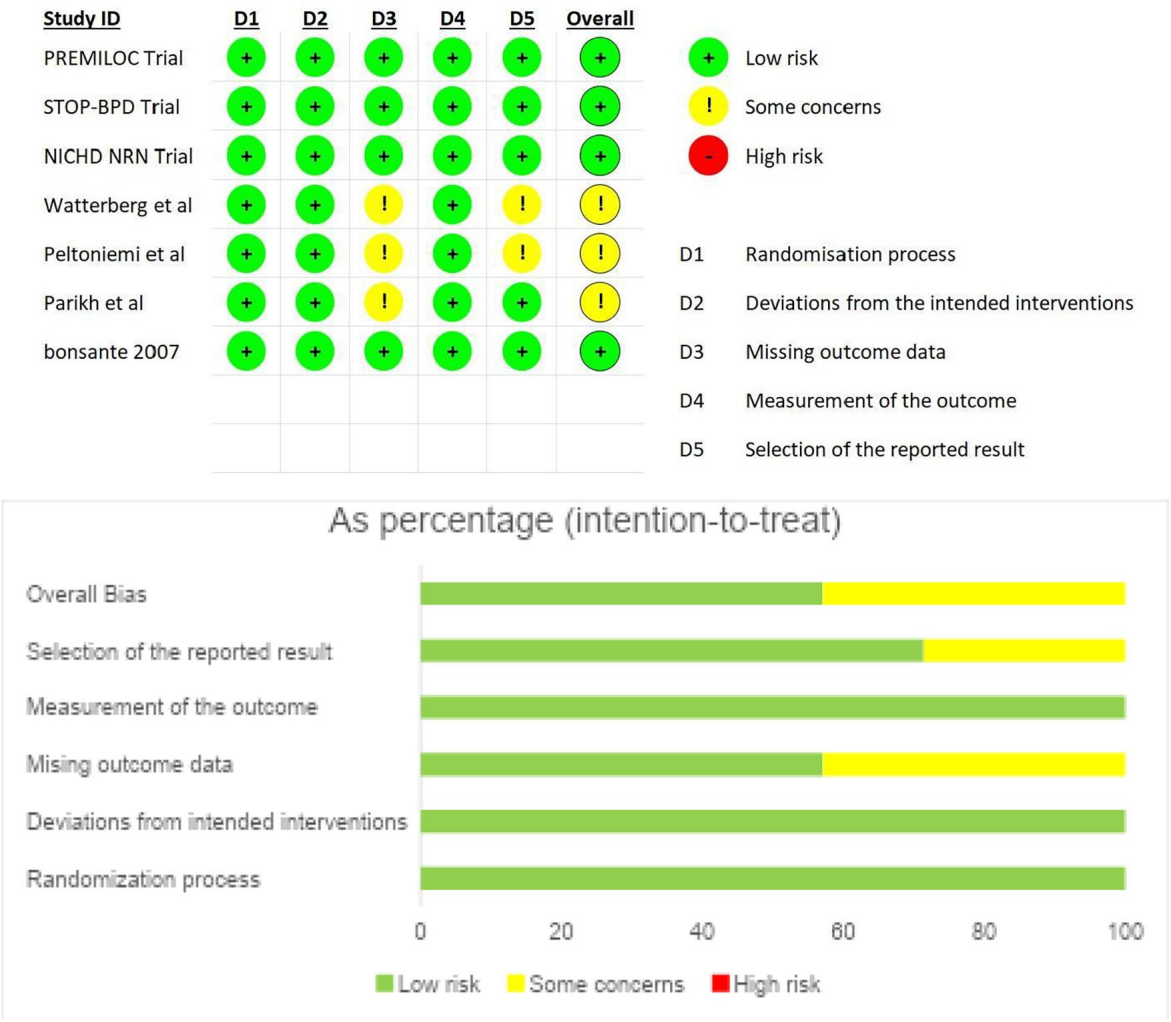
Risk-of-bias assessment using the ROB2 tool for RCTs.

For death or NDI at 2 years, the pooled effect estimate was RR 0.90 (95% CI 0.80–1.01) in the early group and RR 0.97 (95% CI 0.83–1.14) in the late group. When all trials were pooled, the overall effect estimate was RR 0.95 (95% CI 0.88–1.02; *I*² = 0%, 95% CI 0–74.6%; 95% prediction interval 0.86–1.05); the event rates were 53.5% (531/993) and 56.8% (581/1,023) (Figure 2). In addition, we used a DOI plot to detect any possible asymmetry between the study, and a minor asymmetry with an LFK index of −1.17 was found (Supplementary Figure 2).

### 3.4 Secondary outcomes

**Figure 3:**
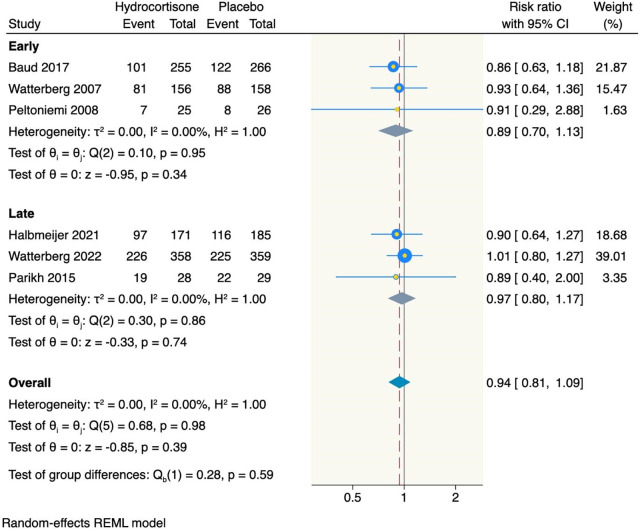
Random-effects model for the composite outcome of death or NDI at 2 years.

**Figure 4:**
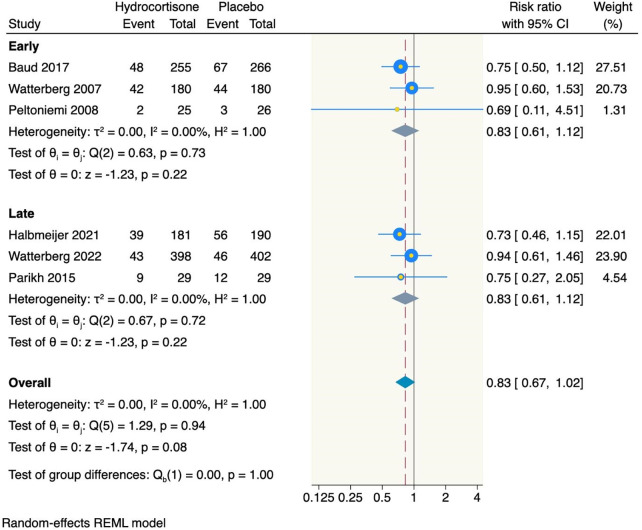
Random-effects model for death at 2 years.

**Figure 5:**
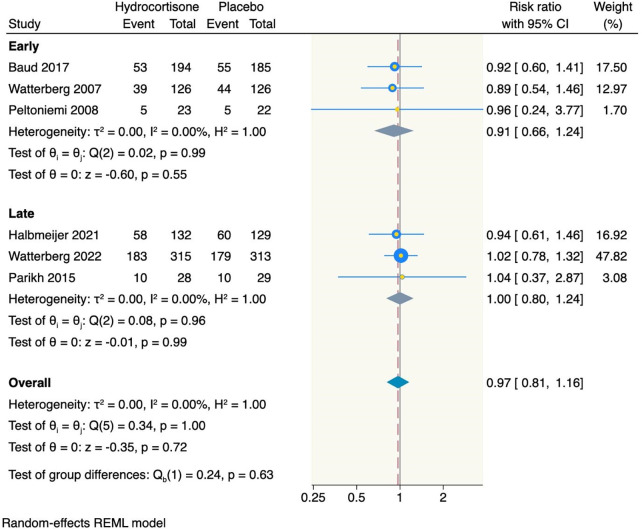
Random-effects model for NDI at 2 years.

Mortality at 2 years was significantly lower among infants who received hydrocortisone (RR 0.82, 95% CI 0.71–0.95, *p* = 0.016; *I*² = 0%; 95% prediction interval 0.65–1.03); the direction was consistent in both timing subgroups, but neither was individually significant (early: RR 0.83, 95% CI 0.57–1.21; late: RR 0.81, 95% CI 0.56–1.18; Q-interaction *p* = 0.85) (Figure 3). NDI at 2 years showed a statistically significant subgroup interaction (*p* < 0.001): early prophylactic administration of hydrocortisone was associated with a reduction in NDI (RR 0.91, 95% CI 0.85–0.97), whereas late treatment was neutral (RR 1.00, 95% CI 0.92–1.10). When all studies were pooled, the overall effect estimate was RR 0.98 (95% CI 0.93–1.04) (Figures 4, 5).

At 2 years, no significant differences were detected in the risk of cerebral palsy in either subgroup (early: RR 1.02, 95% CI 0.43–2.42; late: RR 1.20, 95% CI 0.54–2.68; Q-interaction *p* = 0.55). Hearing impairment showed an overall HK-adjusted reduction (RR 0.56, 95% CI 0.41–0.77), although the DerSimonian–Laird sensitivity estimate yielded a wider interval (RR 0.56, 95% CI 0.30–1.06), so we interpreted this as suggestive only. Visual impairment showed no significant differences in either subgroup (early *k* = 1: RR 0.91, 95% CI 0.55–1.50; late *k* = 2: RR 0.50, 95% CI 0.09–2.70) (Supplementary Figures 3–5).

### 3.5 Exploratory outcomes

At 36 weeks of postmenstrual age, death was significantly reduced with hydrocortisone (RR 0.79, 95% CI 0.67–0.93, *p* = 0.014; early: RR 0.86, 95% CI 0.66–1.11; late: RR 0.71, 95% CI 0.47–1.05; Q-interaction *p* = 0.075). The composite outcome of death/BPD (RR 0.96, 95% CI 0.89–1.04; early: RR 0.96, 95% CI 0.53–1.76, I² = 65%; late: RR 0.97, 95% CI 0.89–1.05) and BPD alone (RR 0.99, 95% CI 0.92–1.07) were not significantly reduced in either subgroup. In addition, we found no significant difference between the study groups at school-age follow-up, up to 5 years, regarding cerebral palsy (RR 1.09, 95% CI: 0.75–1.58, *p* = 0.66), Full-Scale Intelligence Quotient (FSIQ) > 70 (RR 0.84, 95% CI: 0.59–1.21, *p* = 0.35), and change in FSIQ (SMD 0.03, 95% CI: −0.11–0.16, *p* = 0.71), Verbal Scale Intelligence Quotient (VSIQ) (SMD 0.05, 95% CI: −0.08–0.18, *p* = 0.46), or Performance Scale Intelligence Quotient (PSIQ) (SMD −0.11, 95% CI: −0.58–0.36, *p* = 0.64) (Supplementary Appendix).

### 3.6 Sensitivity analyses

In leave-one-out analyses, the non-significant findings remained consistent: pooled estimates for death or NDI (RR range 0.90–0.96), NDI alone (0.93–0.99), BPD alone (0.98–1.01), and the composite outcome of death or BPD at 36 weeks of postmenstrual age (0.95–0.97) were unchanged in direction, remained non-significant, and showed low heterogeneity (*I*² ≤ 8%). The reduction in 2-year mortality (RR 0.82, 95% CI 0.71–0.95) lost significance when either the Baud 2017 or Halbmeijer 2021 trial was omitted (RR 0.85; *p* = 0.06). Restricting the analysis to the four trials enrolling ≥100 infants gave consistent results for every outcome (e.g., death or NDI: RR 0.95, 95% CI 0.85–1.06), with mortality no longer significant (RR 0.83, 95% CI 0.66–1.04). These findings suggest that the neurodevelopmental and respiratory findings were not driven by any single trial or smaller pilot studies; however, the mortality signal should be interpreted with caution (Supplementary Table 2).

## 4 Discussion

In our meta-analysis of randomized controlled trials, we evaluated the effects of postnatal hydrocortisone in extremely preterm infants. We found no statistically significant difference in the composite outcome of death or NDI at 2 years compared with placebo, and no evidence of increased neurotoxicity risk across major neurologic and sensory endpoints.

Clinically, these findings address the primary barrier to steroid use in this population: the historical experience with early systemic dexamethasone, where respiratory benefits were balanced by concern for adverse neurodevelopmental outcomes (28–30). Our pooled results provide evidence that hydrocortisone does not demonstrate the same pattern of harm.

The key question is whether clinicians can support lung development and facilitate respiratory stability without increasing long-term disability. In our analysis, overall event rates for death/NDI were numerically lower in the hydrocortisone group; however, the pooled estimates showed no significant difference, with similarly neutral findings for NDI alone. We should interpret these interpreted precisely. While we did not detect an increased risk of clinically recognized neurodevelopmental harm, this does not exclude the possibility of a small risk, particularly for rarer outcomes. Therefore, extending follow-up into school age and using complementary objective measures, such as MRI metrics, will be critical for a true neurotoxicity narrative.

The timing-stratified analysis identified a significant interaction for NDI at 2 years: early prophylactic hydrocortisone was associated with a lower risk of NDI, whereas late treatment showed no effect. This pattern is biologically reasonable, suggesting that anti-inflammatory effects may be most beneficial during the earliest postnatal period and supporting analysis by treatment timing. At school age, two early-intervention trials reported a numerically five times higher rate of cerebral palsy in the hydrocortisone group, based on only 7 versus 1 events among 118 children. Because neither trial was individually significant and the pooled estimate is unstable, this finding should be considered hypothesis-generating rather than evidence of harm until confirmed in larger long-term studies.

A central harm concern is the potential risk of cerebral palsy and neurocognitive impairment. Across trials reporting 2-year follow-up outcomes, hydrocortisone was not associated with significantly different rates of cerebral palsy, hearing impairment, or visual impairment compared with placebo. This aligns with long-term follow-up findings from the major randomized programs: early low-dose prophylactic hydrocortisone trials did not show worse overall neurodevelopmental outcomes, and follow-up from later-treatment trials similarly has not demonstrated a clear signal of neurodevelopmental harm, even with higher cumulative dosing in more critically ill infants.

School-age outcomes are particularly valuable because milder cognitive, executive function, and academic difficulties may not be fully captured at 18–26 months. Available school-age follow-up data show no difference in functional impairment between the hydrocortisone and placebo groups, despite a high overall burden of impairment typical of extremely preterm populations. Finally, neuroimaging evidence provides an objective link between exposure and potential mechanisms of brain injury; available MRI substudies do not suggest worsening of brain abnormality scores or reductions in regional brain volumes at term-equivalent age among infants treated with hydrocortisone (31, 32). These clinical outcomes support the conclusion that hydrocortisone does not appear to increase the risk of neurotoxicity.

To strengthen the neurotoxicity argument, neuroimaging data provide an objective link between exposure and mechanisms of potential brain injury. In the STOP-BPD MRI study (21), hydrocortisone initiated in the second week after birth was not associated with differences in brain abnormality scores or regional brain volumes at term-equivalent age compared with placebo. While such imaging outcomes do not replace functional endpoints, they support the clinical observation that hydrocortisone exposure does not appear to impair early brain growth or increase major brain injury markers.

It is clinically important to acknowledge the respiratory side of the balance. In our pooled analyses at 36 weeks of postmenstrual age, hydrocortisone was not associated with any significant reduction in the composite outcome of death or BPD, nor in BPD alone. However, there was a borderline signal toward reduced mortality at 36 weeks in the pooled estimate. This pattern mirrors the nuance seen in large late-treatment trials, where the primary outcome of death or BPD may remain unchanged, while selected secondary respiratory outcomes and early mortality trends move in a favorable direction, with long-term neurodevelopmental outcomes remaining neutral.

In contrast, early prophylactic low-dose hydrocortisone regimens have shown clearer improvement in survival without BPD at 36 weeks, supporting a timing-dependent treatment effect. Conceptually, earlier modulation of inflammation may be better able to alter the course toward BPD than later “rescue” therapy once lung injury is established. Importantly, this potential respiratory advantage has not been accompanied by an apparent neurodevelopmental penalty on follow-up, which is the key clinical tradeoff clinicians seek to avoid.

A harm-focused discussion should explicitly distinguish between (1) feared neurotoxicity and (2) expected systemic steroid adverse effects. Across major trials, the incidence of serious adverse events was broadly similar between the hydrocortisone and placebo groups; however, certain expected physiological side effects were common with hydrocortisone, depending on the treatment regimen and population. In the STOP-BPD trial (late, high cumulative dose), hyperglycemia requiring insulin occurred more frequently in the hydrocortisone group than in the placebo group (12). In the NRN trial published in the *NEJM*, hypertension requiring medication was more frequent in the hydrocortisone group (27). These findings support a practical clinical recommendation: when hydrocortisone is used, glucose levels and blood pressure should be monitored closely, particularly in late-treatment regimens and in infants with other risk factors for metabolic instability.

Gastrointestinal perforation has historically been a major safety concern with early postnatal steroids, especially in combination with non-steroidal anti-inflammatory drugs used for patent ductus arteriosus (PDA) management. Even when a given meta-analysis does not focus on this endpoint, we want to acknowledge it and frame it as a preventable risk through cointervention awareness rather than neurotoxicity. The PREMILOC trial did not demonstrate a significant difference in overall gastrointestinal perforation, although neonatal practice and cotreatments vary across eras and settings (11). Contemporary syntheses emphasize that timing, comedications, and patient selection are central to balancing benefit and harm.

A consistent trend across the included trials is that “hydrocortisone” should not be viewed as a uniform exposure. Early prophylactic regimens are typically administered at lower doses during a period of adrenal immaturity and early inflammatory programming, whereas regimens initiated after 7 days of age are used in ventilator-dependent infants with evolving or established lung injury and often involve higher cumulative doses. In addition, definitions of BPD (physiologic vs. clinical vs. severity-graded) and NDI (different Bayley versions, IQ thresholds, and cerebral palsy classification cutoffs) vary across trials, which can reduce the sensitivity to detect subtle neurodevelopmental shifts and complicate pooled comparisons. Notably, BSID-III generally yields higher scores than BSID-II and classifies fewer infants as impaired at the same cutoff; therefore, the shift between editions across trials may affect reported rates of impairment and limit comparability between studies.

Our pooled long-term findings are broadly consistent with the largest randomized trials. Across these studies, the overall message is reassuring for neurodevelopment: follow-up assessments do not demonstrate a clear increase in neurodevelopmental impairment, cerebral palsy, or major sensory disability among infants exposed to hydrocortisone compared with placebo. This alignment across large trials strengthens the interpretation that hydrocortisone, as used in modern regimens, does not appear to carry a detectable neurotoxicity signal.

In early prophylactic low-dose strategies (by PREMILOC-type regimens), the evidence tends to show a clearer respiratory benefit, most notably an improvement in survival without BPD at 36 weeks, while maintaining reassuring neurodevelopmental outcomes at 2 years and beyond. Clinically, this supports the concept that earlier modulation of inflammation may be more effective at shifting the disease course toward less BPD, without introducing a measurable long-term neurodevelopmental issue.

In later “treatment” strategies used for ventilator-dependent infants with evolving or established lung injury (STOP-BPD-type regimens), the primary composite outcome of death or BPD at 36 weeks is often not significantly improved, and the overall long-term neurodevelopmental signal remains neutral. However, these later regimens more consistently demonstrate the expected short-term physiological side effects (e.g., metabolic instability, such as hyperglycemia), emphasizing the need for careful monitoring even when neurodevelopmental safety appears reassuring.

Similarly, in large multicenter late-hydrocortisone programs (such as NRN-type regimens), results typically show no major improvement in the primary composite outcomes at 36 weeks or in survival without moderate/severe NDI at 2 years, with school-age functional outcomes remaining comparable between groups. These trials also highlight that some expected steroid-related adverse effects (such as hypertension requiring treatment) can occur, reinforcing the clinical distinction between “neurotoxicity” concerns and predictable systemic steroid effects that are manageable with monitoring and supportive care.

Where our overall pooled respiratory results may appear less “positive” than the early prophylactic trials is largely explained by mixing across clinical and methodological factors: when early low-dose prophylactic studies are combined with late, higher-cumulative-dose treatment studies in more severely ill infants, the magnitude of any respiratory benefit can be diluted. This should not be viewed as a contradiction; rather, it suggests that the respiratory efficacy of hydrocortisone is likely timing- and phenotype-dependent, whereas its neurodevelopmental safety appears more consistent across treatment regimens and study settings.

### 4.1 Strengths and limitations

Our study has several strengths: this is the only meta-analysis to assess both the short- and long-term efficacy of hydrocortisone for BPD. Additional strengths include the restriction to high-quality randomized controlled trials, the inclusion of long-term follow-up reports, and the broadly consistent findings across multiple neurodevelopmental domains.

However, we acknowledge some limitations. Neurodevelopmental follow-up is vulnerable to attrition, and cross-over or open-label steroid exposure in some trials may attenuate true between-group differences. Follow-up rates were high at 2 years but lower at school age; therefore, attrition bias, especially at later ages, warrants caution when interpreting the results. In addition, rare outcomes (such as cerebral palsy and severe sensory impairment) remain underpowered across the overall evidence base, meaning that small-to-moderate risk differences cannot be fully excluded. Although the pooled studies were statistically homogeneous, this should not be interpreted as the absence of true between-study heterogeneity. Clinical heterogeneity exists across our included trials with respect to the timing of intervention (early prophylactic vs. late treatment), dosing regimens, study population (all preterm vs. ventilator-dependent infants), and outcome definitions (NDI assessed with Bayley-II vs. Bayley-III and cutoffs ranging from <70 to <85).

### 4.2 Clinical implications

Taken together, the best available evidence from randomized trials suggests that postnatal hydrocortisone is not associated with an increased risk of neurodevelopmental impairment or other major neurotoxicity signals compared with placebo; however, expected systemic adverse effects of steroid therapy should be anticipated and monitored.

## 5 Conclusion

In extremely preterm infants, postnatal hydrocortisone was not associated with a significant improvement in the composite outcome of death or neurodevelopmental impairment at 2 years, nor was it associated with an increased risk of cerebral palsy or major sensory impairment. Overall respiratory benefits at 36 weeks of postmenstrual age were not consistently demonstrated. Larger studies with standardized definitions and longer-term follow-up are needed to better identify which infants are most likely to derive the greatest net benefit.

## Data Availability

The original contributions presented in the study are included in the article/Supplementary Material, further inquiries can be directed to the corresponding authors.
