# Supplementary material for: Hydrocortisone for prevention or treatment of bronchopulmonary dysplasia: long-term neurodevelopmental safety and efficacy—a meta-analysis of randomized clinical trials
Source: Front Pediatr. 2026 Jun 24;14:1851870. doi: 10.3389/fped.2026.1851870 (PMC13341419; doi:10.3389/fped.2026.1851870)

**Supplementary Appendix**

| **Content** | **Page** |
| --- | --- |
| **Table S1**. Detailed search strategy on different databases. | **2** |
| **Figure S1**. DOI plot with LFK index of Composite of Death or NDI at 2 years. | **3** |
| **Figure S2.** Random-effect model of Cerebral palsy at 2 years. | **4** |
| **Figure S3.** Random-effect model of Hearing Impairment at 2 years. | **5** |
| **Figure S4.** Random-effect model of Visual Impairment at 2 years. | **6** |
| **Figure S5.** Random-effect model of Death or BPD at 36 weeks. | **7** |
| **Figure S6.** Random-effect model of BPD at 36 weeks. | **8** |
| **Figure S7.** Random-effect model of Death 36 weeks. | **9** |
| **Figure S8.** Random-effect model of CP at school age. | **10** |
| **Figure S9.** Random-effect model of FSIQ < 70. | **11** |
| **Figure S10.** Random-effect model of VSIQ. | **12** |
| **Figure S11.** Random-effect model of PSIQ. | **13** |
| **Figure S11.** Random-effect model of FSIQ. | **14** |

**Table S1**. Detailed search strategy on different databases.

| Data Base | Search term | Field/Filters | Date | Result |
| --- | --- | --- | --- | --- |
| PubMed | (hydrocortisone OR cortisol) AND (preterm OR pre mature OR newborn OR neonate OR infant OR low birth weight OR VLBW OR ELBW) AND (bronchopulmonary dysplasia OR BPD OR chronic lung disease) | All Fields, English | ^10th^ of February 2025 | 290 |
| Scopus | (hydrocortisone OR cortisol) AND (preterm OR pre mature OR newborn OR neonate OR infant OR low birth weight OR VLBW OR ELBW) AND (bronchopulmonary dysplasia OR BPD OR chronic lung disease) | Title, abstract, keyword, English | ^10th^ of February 2025 | 68 |
| WOS | (hydrocortisone OR cortisol) AND (preterm OR pre mature OR newborn OR neonate OR infant OR low birth weight OR VLBW OR ELBW) AND (bronchopulmonary dysplasia OR BPD OR chronic lung disease) | All Fields, English | ^10th^ of February 2025 | 419 |
| Cochrane central | (hydrocortisone OR cortisol) AND (preterm OR pre mature OR newborn OR neonate OR infant OR low birth weight OR VLBW OR ELBW) AND (bronchopulmonary dysplasia OR BPD OR chronic lung disease) | All Fields, English | ^10th^ of February 2025 | 117 |

**Figure S1**. DOI plot with LFK index of Composite of Death or NDI at 2 years.


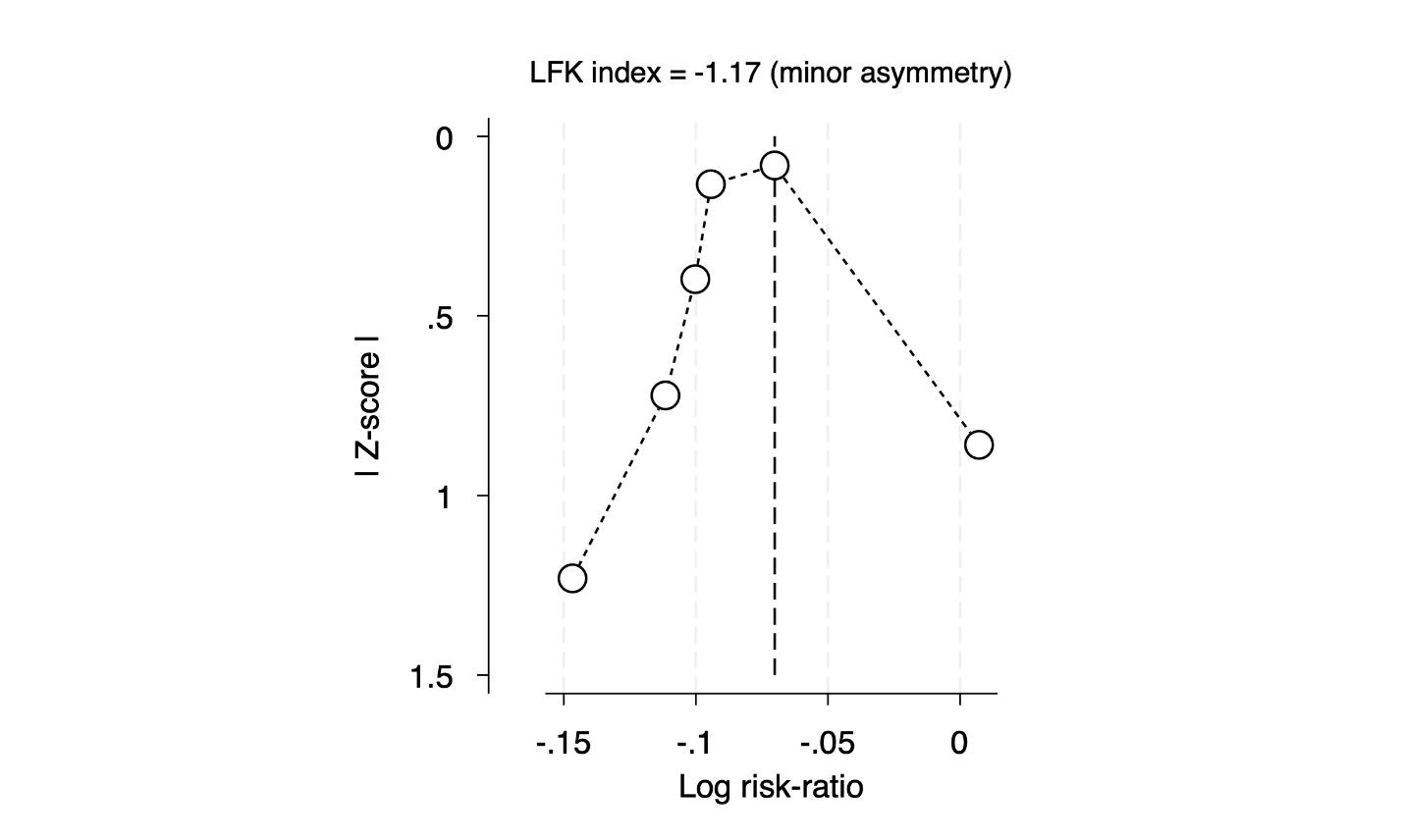


**Figure S2.** Random-effect model of Cerebral palsy at 2 years.

**
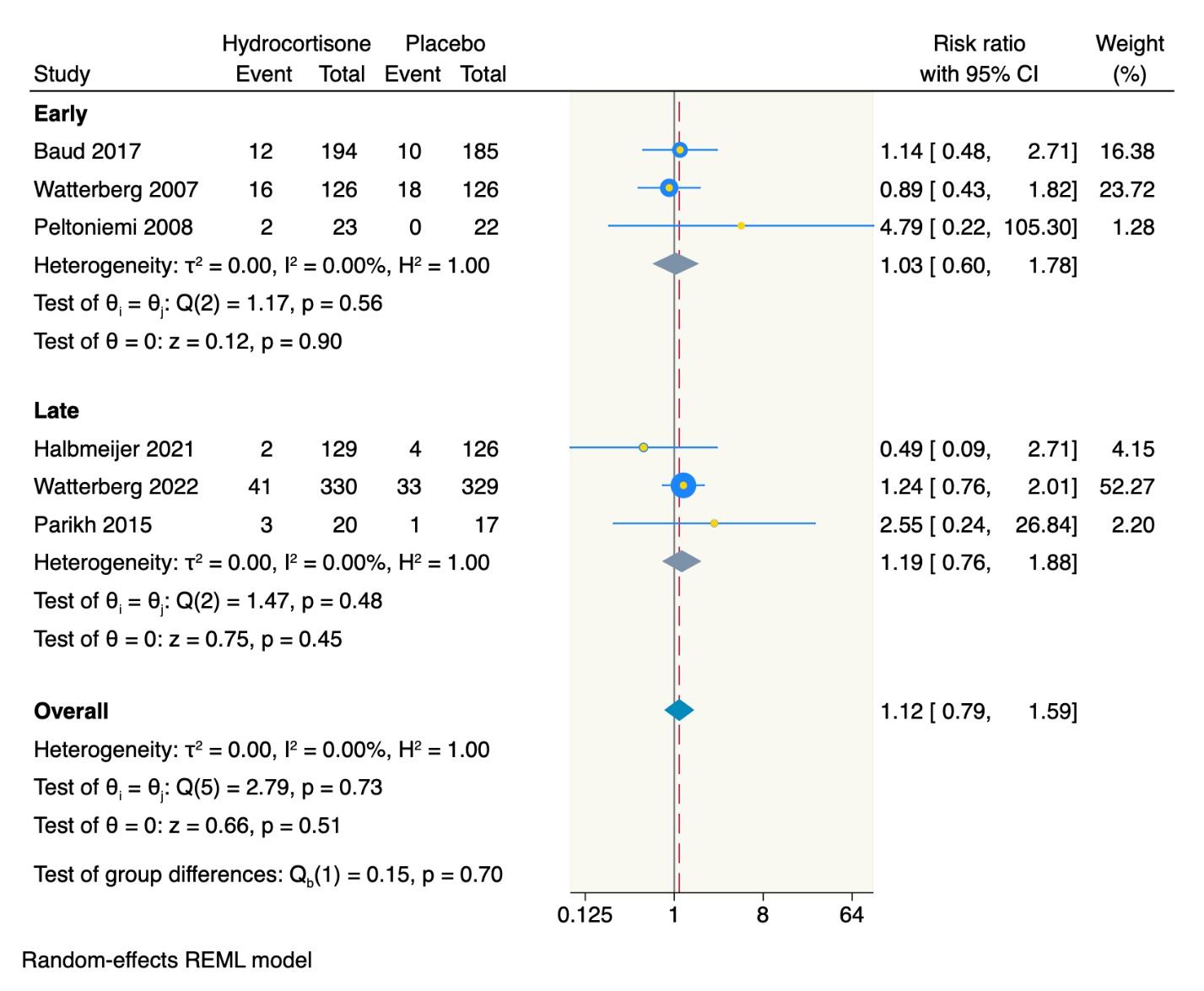
**

**Figure S3.** Random-effect model of Hearing Impairment at 2 years.


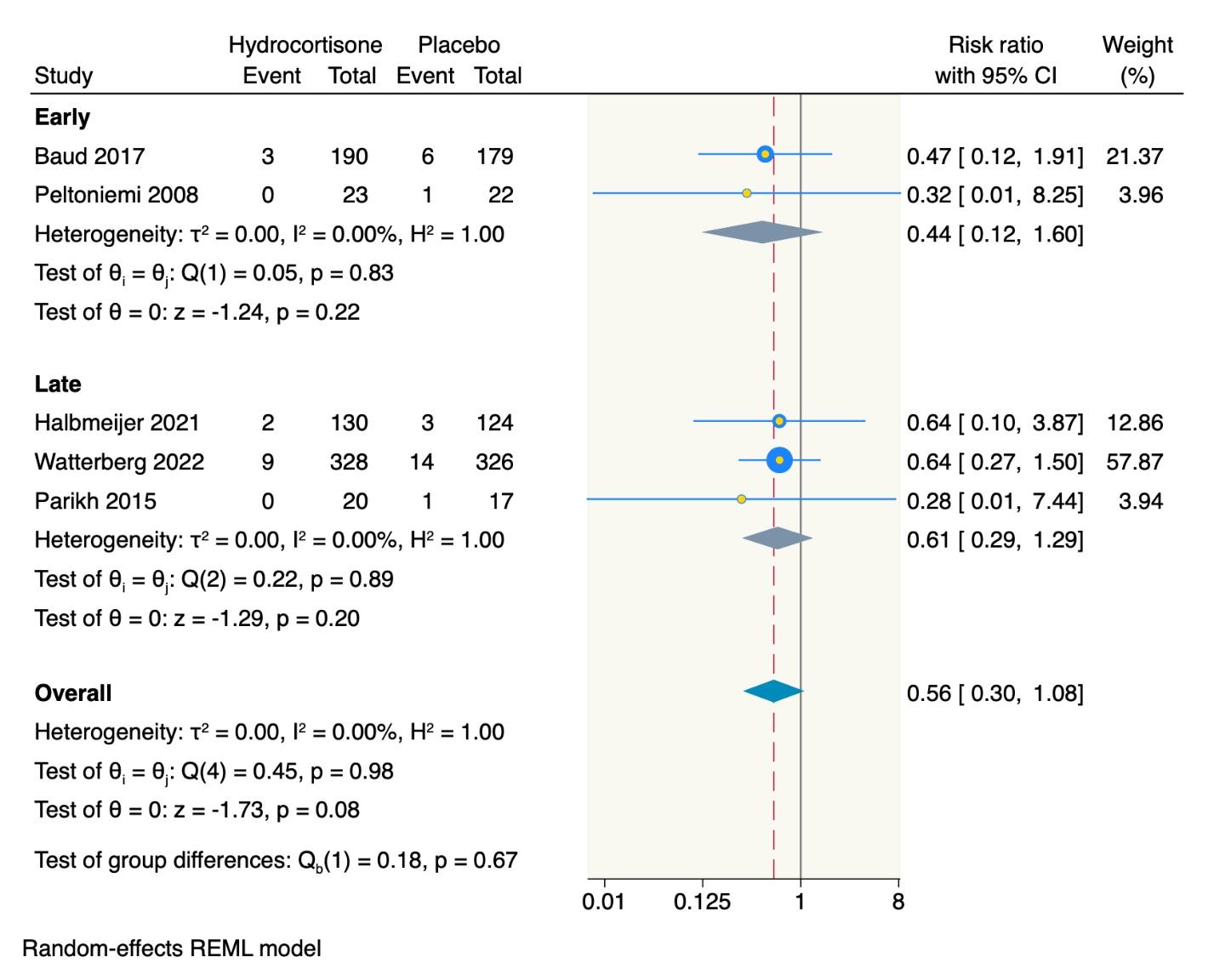


**Figure S4.** Random-effect model of Visual Impairment at 2 years.


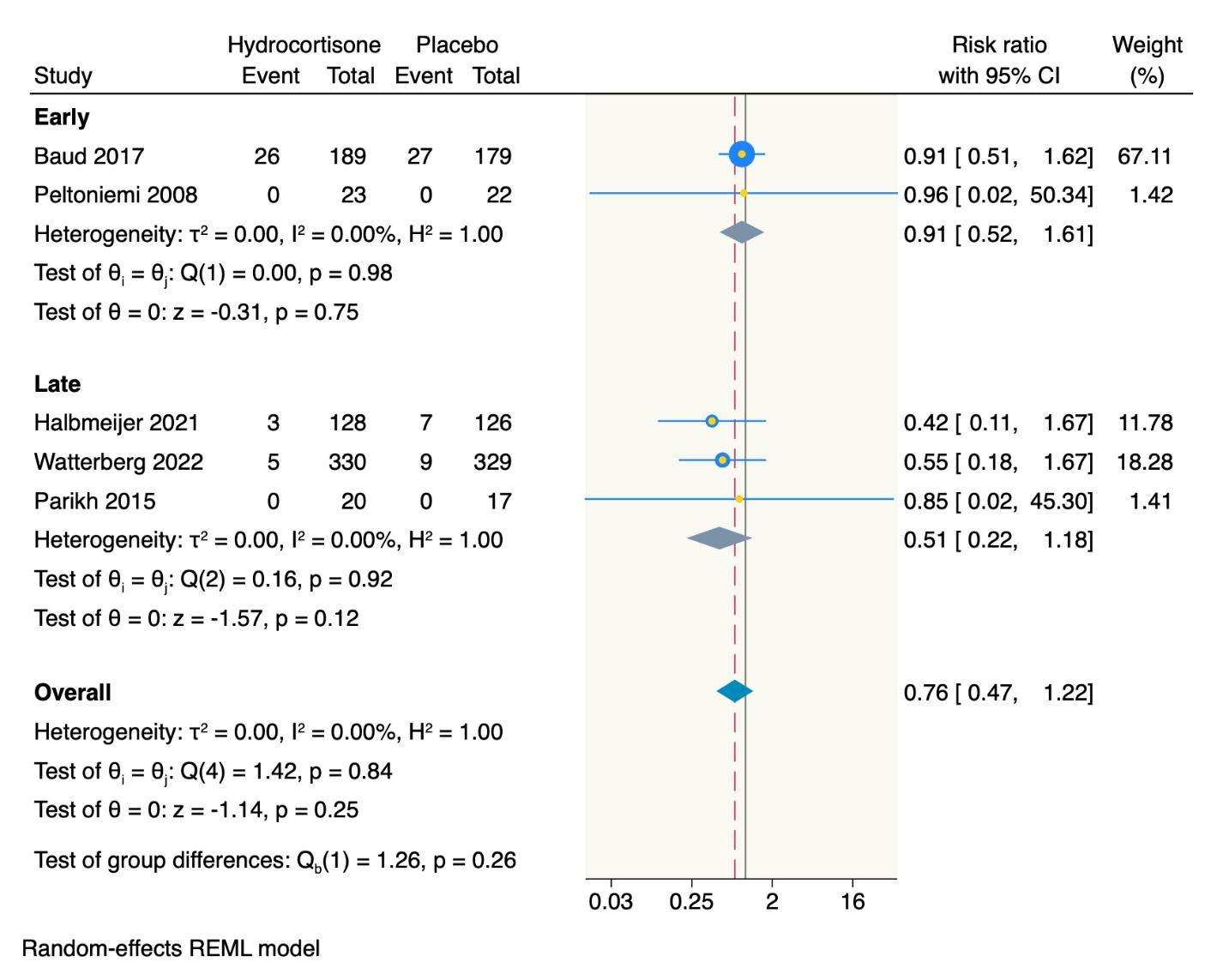


**Figure S5.** Random-effect model of Death or BPD at 36 weeks.


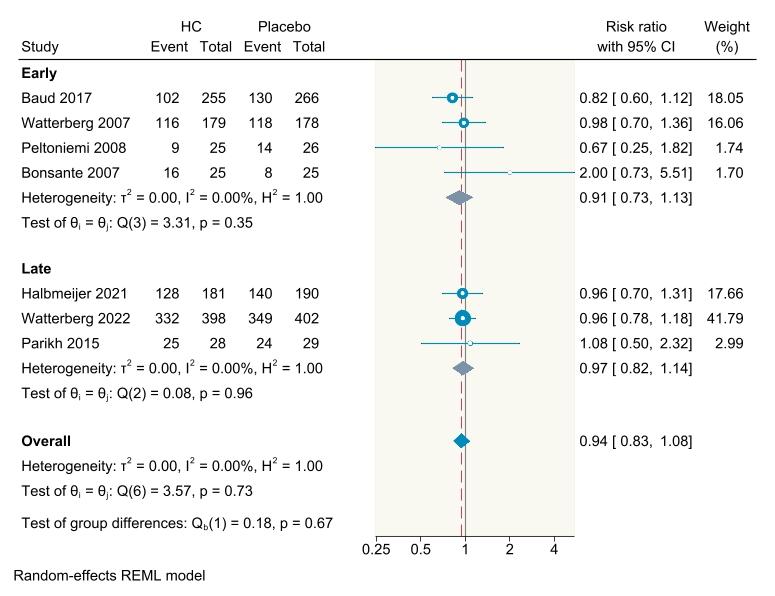


**Figure S6.** Random-effect model of BPD at 36 weeks.


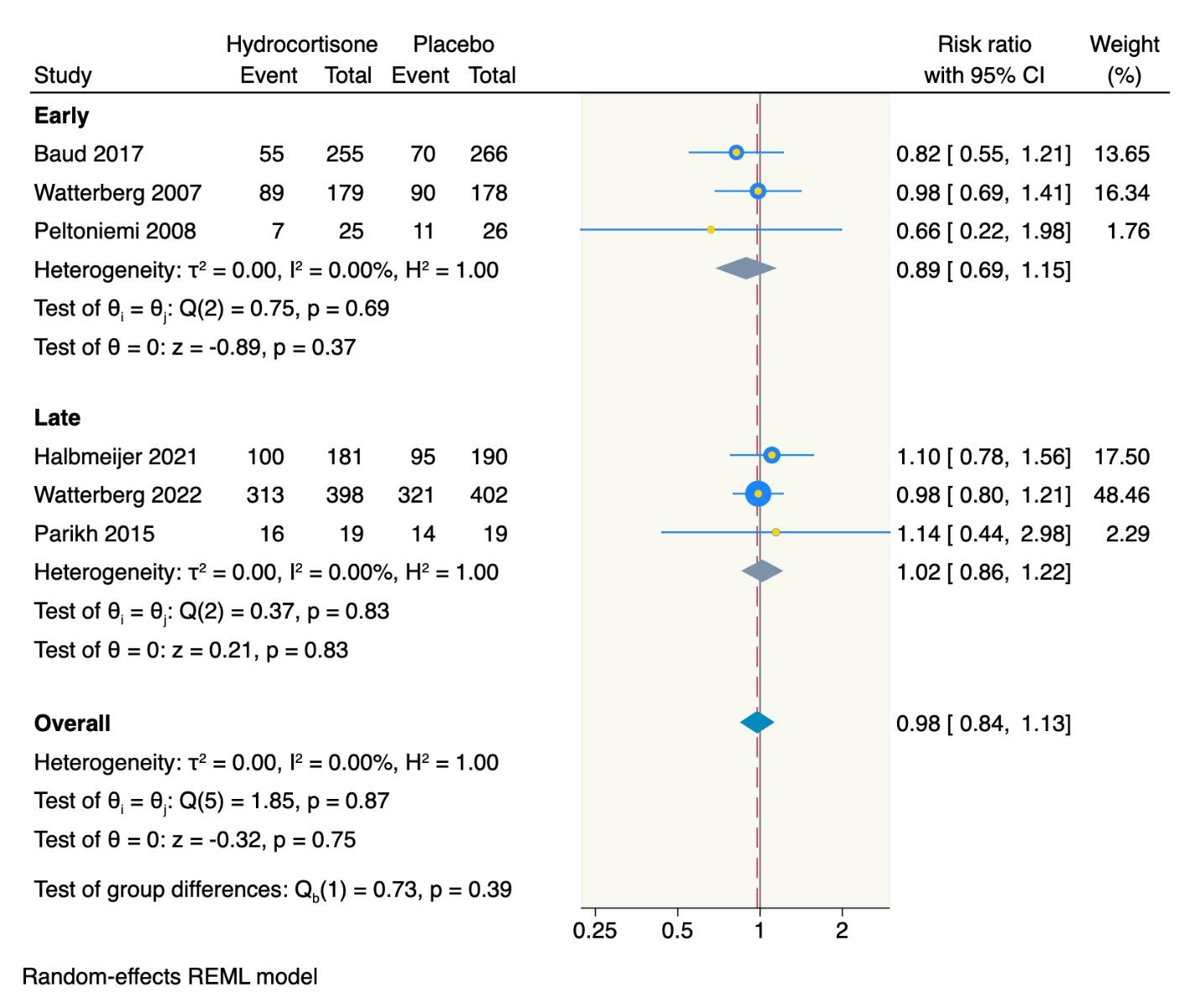


**Figure S7.** Random-effect model of Death 36 weeks.


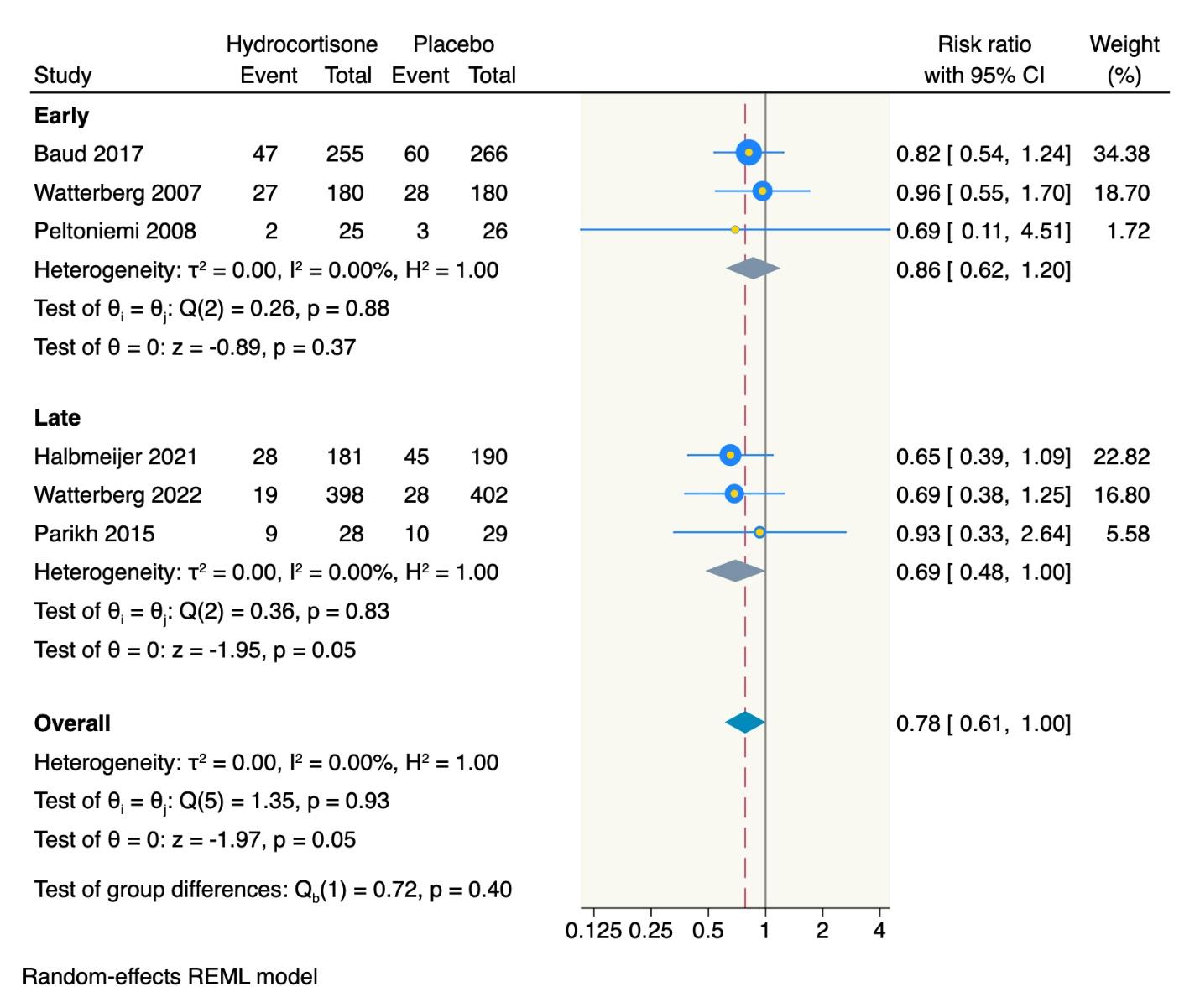


**Figure S8.** Random-effect model of CP at school age.
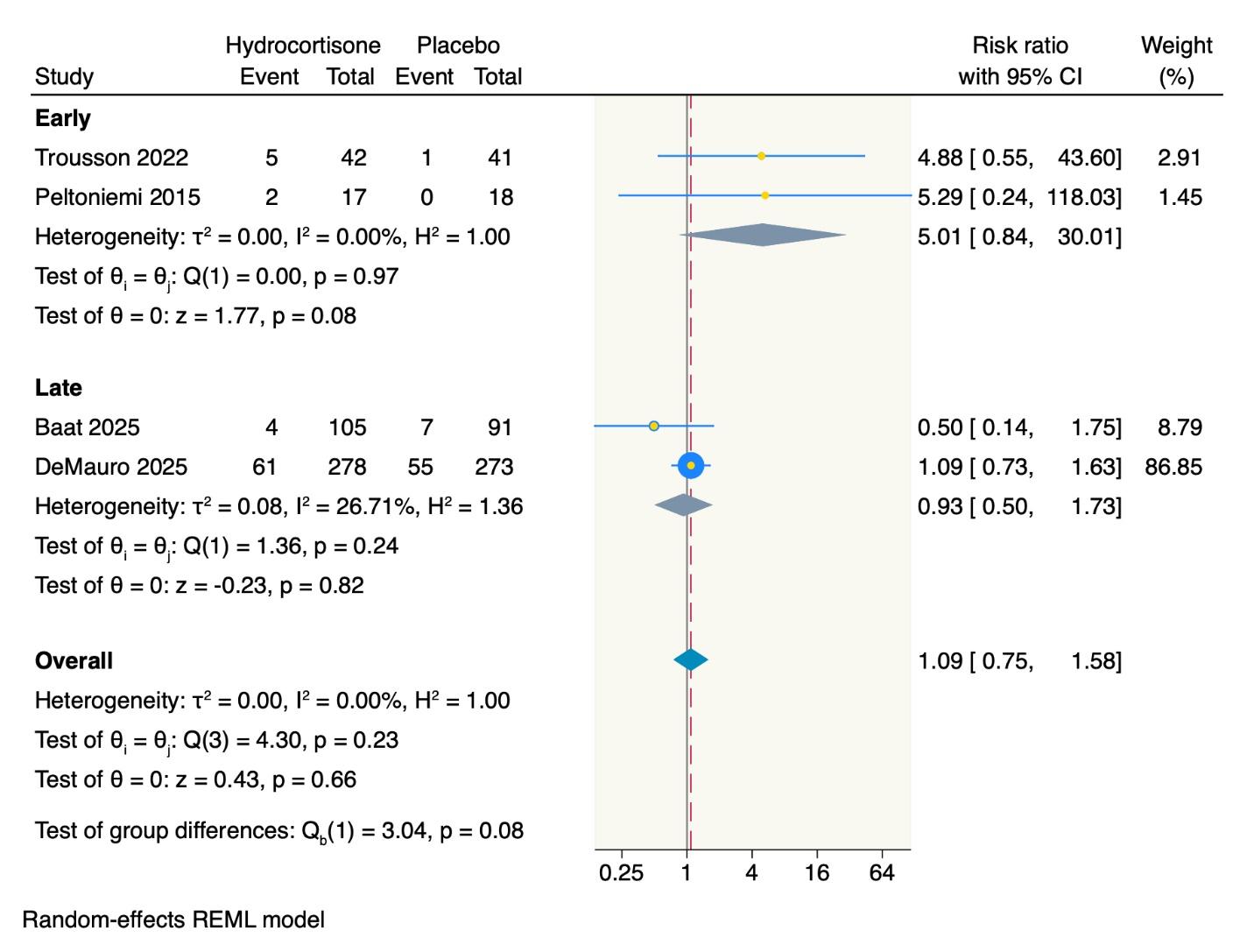
.

**Figure S9.** Random-effect model of FSIQ < 70.


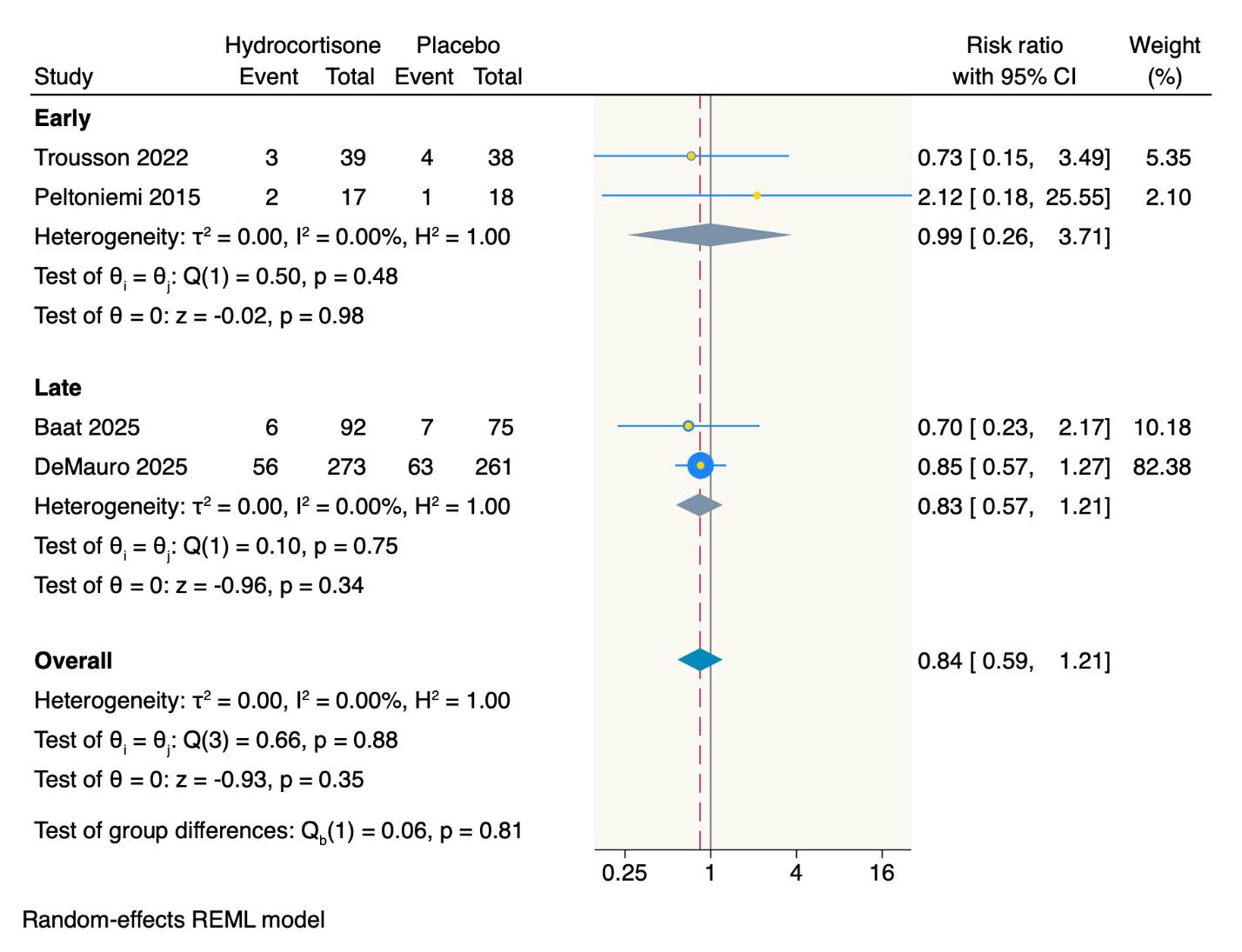


**Figure S10.** Random-effect model of VSIQ.


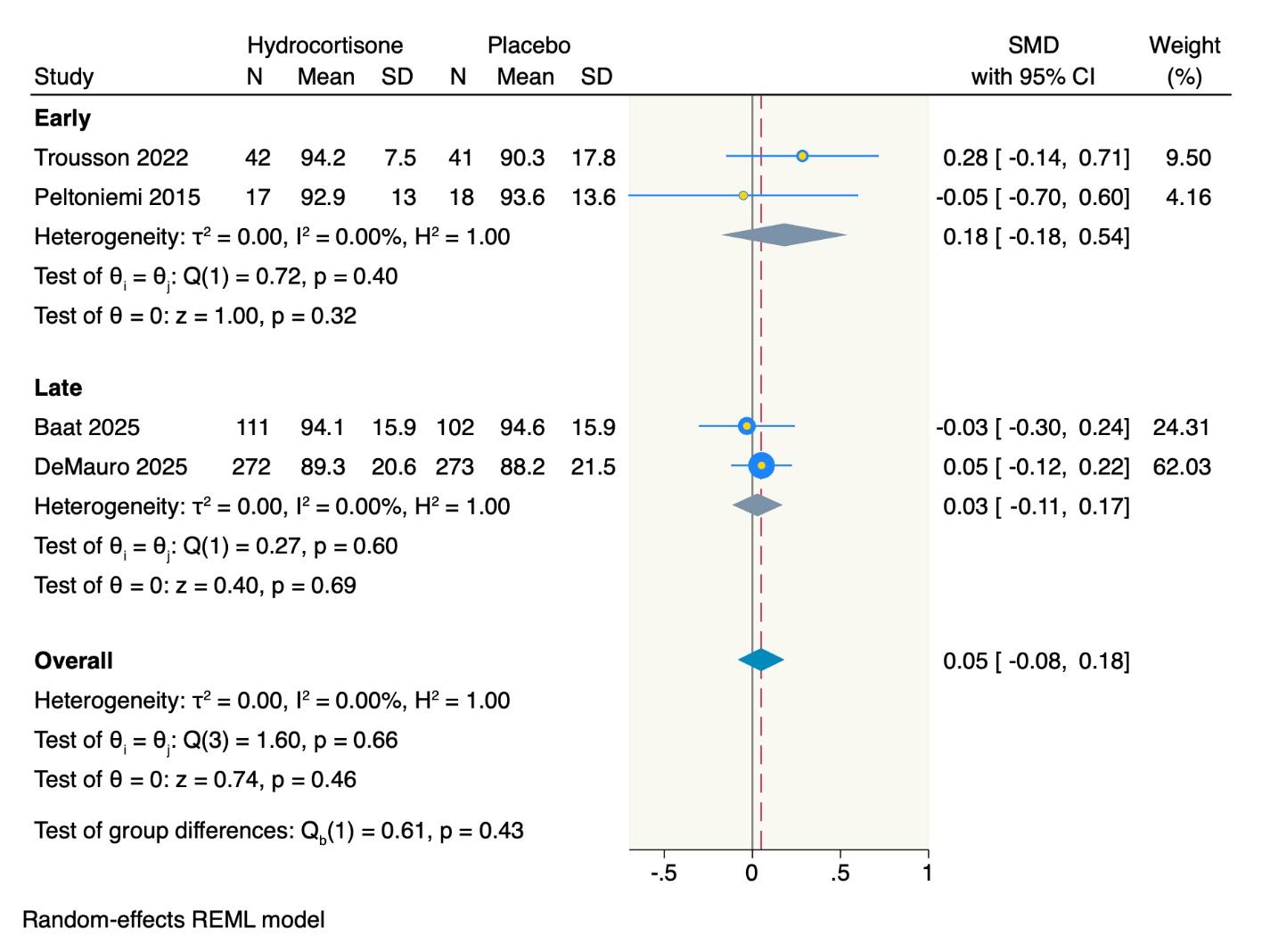


**Figure S11.** Random-effect model of PSIQ.
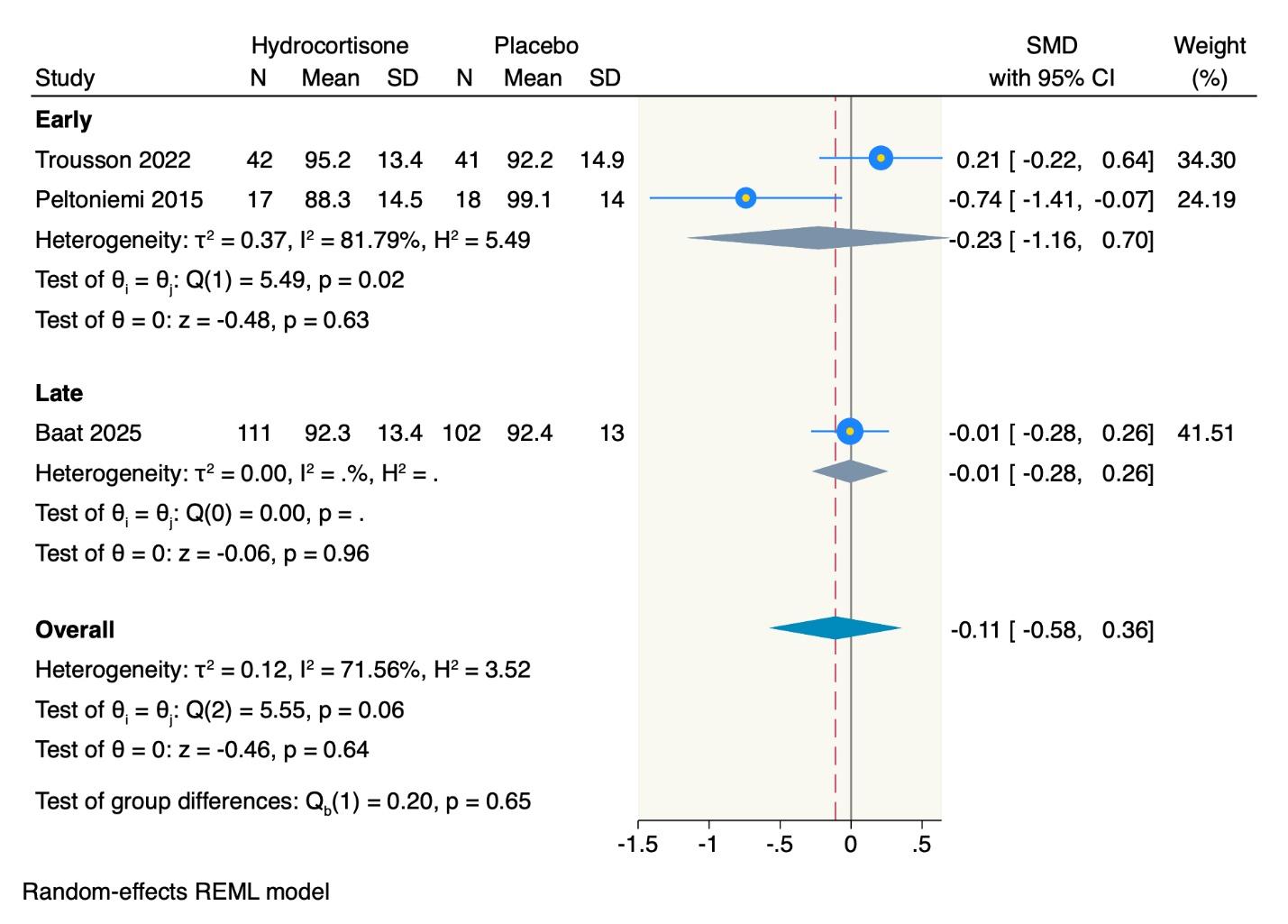
.

**Figure S11.** Random-effect model of FSIQ.


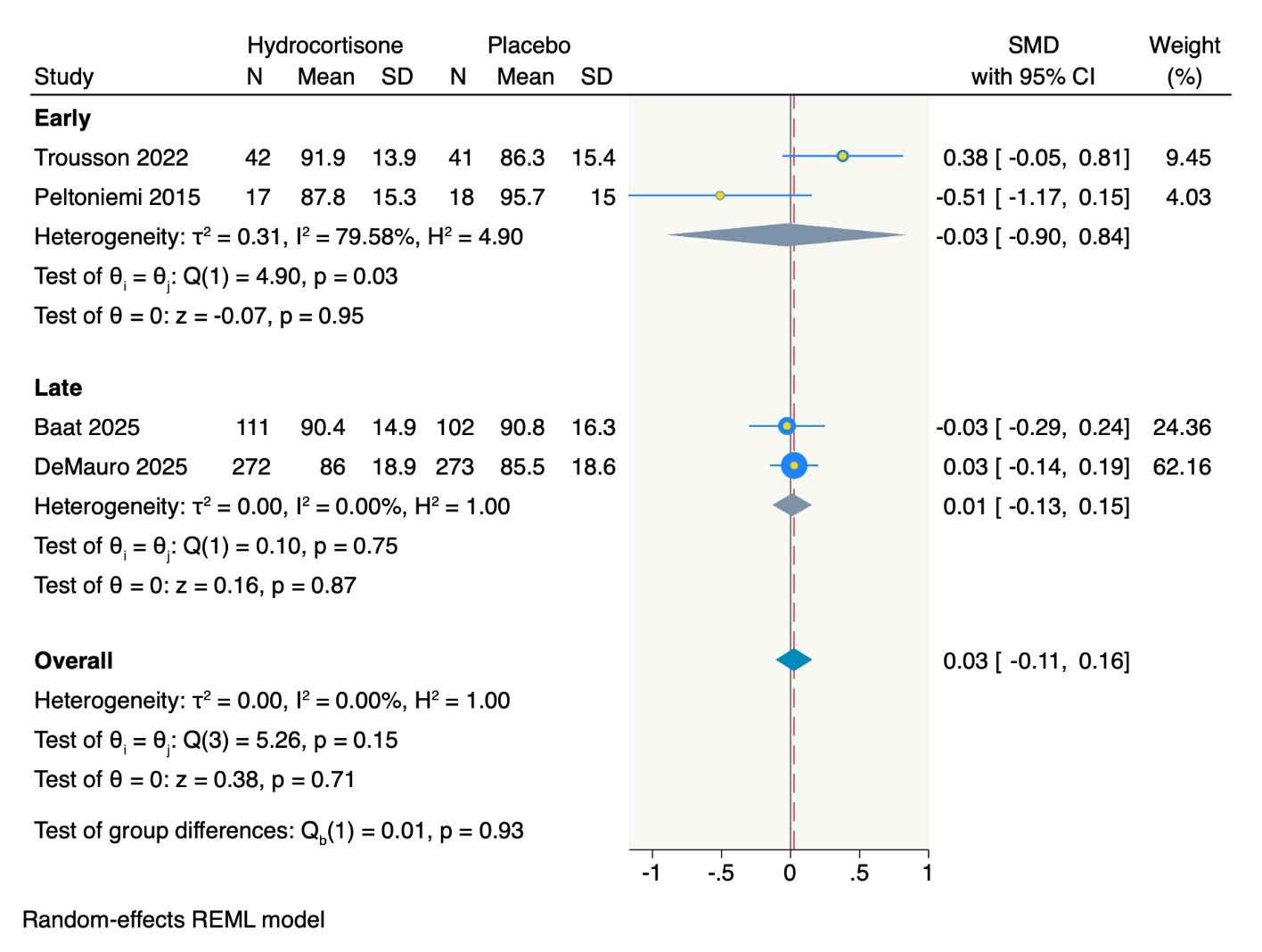

Supplement: Supplementary file 1 [file Supplementaryfile1.docx]
